# Rattlesnake Crotalphine Analgesic Active on Tetrodotoxin-Sensitive Na^+^ Current in Mouse Dorsal Root Ganglion Neurons

**DOI:** 10.3390/toxins16080359

**Published:** 2024-08-15

**Authors:** Aurélie Antunes, Philippe Robin, Gilles Mourier, Rémy Béroud, Michel De Waard, Denis Servent, Evelyne Benoit

**Affiliations:** 1 Département Médicaments et Technologies pour la Santé (DMTS), Institut des Sciences du Vivant Frédéric Joliot, Université Paris-Saclay, CEA, Service d’Ingénierie Moléculaire pour la Santé (SIMoS), EMR CNRS/CEA 9004, F-91191 Gif-sur-Yvette, France; aurelie.antunes@cea.fr (A.A.); philippe.robin@cea.fr (P.R.); mourier.g@gmail.com (G.M.); denis.servent@cea.fr (D.S.); 2 Smartox Biotechnology, F-38120 Saint-Egrève, France; remy.beroud@smartox-biotech.com (R.B.); michel.dewaard@univ-nantes.fr (M.D.W.); 3 L’Institut du Thorax, INSERM, CNRS, Université de Nantes, F-44007 Nantes, France; 4 LabEx “Ion Channels, Science and Therapeutics”, F-06560 Valbonne, France

**Keywords:** cell viability assay, crotalphine, electrophysiology, mouse dorsal root ganglion neurons, rattlesnake toxin, tetrodotoxin-sensitive Na^+^ channels

## Abstract

Crotalphine is an analgesic peptide identified from the venom of the South American rattlesnake *Crotalus durissus terrificus*. Although its antinociceptive effect is well documented, its direct mechanisms of action are still unclear. The aim of the present work was to study the action of the crotalid peptide on the Na_V_1.7 channel subtype, a genetically validated pain target. To this purpose, the effects of crotalphine were evaluated on the Na_V_1.7 component of the tetrodotoxin-sensitive Na^+^ current in the dorsal root ganglion neurons of adult mice, using the whole-cell patch-clamp configuration, and on cell viability, using propidium iodide fluorescence and trypan blue assays. The results show that 18.7 µM of peptide inhibited 50% of the Na^+^ current. The blocking effect occurred without any marked change in the current activation and inactivation kinetics, but it was more important as the membrane potential was more positive. In addition, crotalphine induced an increase in the leakage current amplitude of approximately 150% and led to a maximal 31% decrease in cell viability at a high 50 µM concentration. Taken together, these results point out, for the first time, the effectiveness of crotalphine in acting on the Na_V_1.7 channel subtype, which may be an additional target contributing to the peptide analgesic properties and, also, although less efficiently, on a second cell plasma membrane component, leading to cell loss.

## 1. Introduction

**Figure 1:**
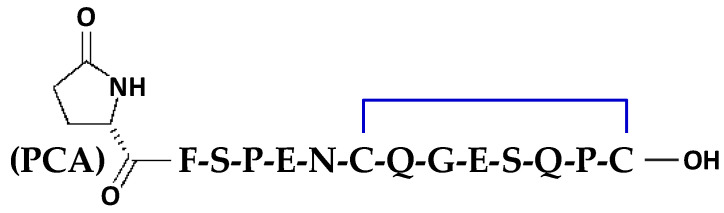
Chemical structure of crotalphine. C: cysteine; E: glutamic acid; F: phenylalanine; G: glycine; N: asparagine; P: proline; Q: glutamine; S: serine; and PCA: pyroglutamic acid (non-standard amino acid).

The observed absence of pain, or the relatively mild pain, felt by victims of *Crotalus durissus* bites, compared to other snakebites, raised the suspicion of a possible analgesic activity of the venom of these South American rattlesnakes [1,2,3]. Hence, the potent and long-lasting antinociceptive effect of the venom of *Crotalus durissus terrificus* [4], well known in human beings since 1934 [5], was experimentally demonstrated in mice approximately 60 years later, suggesting an endorphin-like antinociceptive activity endowed by at least one substance present in this venom [6]. This substance was then purified and identified as crotalphine, a peptide consisting of 14 amino acids with a disulfide bond between Cys^7^ and Cys^14^ (Figure 1) and reported to differ from crotoxin, the main toxic component of crotalid venom [7], and from crotamine, the most abundant protein in this venom [8]. However, the crotalphine sequence is identical to the γ-chain sequence of crotapotin, the chaperon protein subunit of the non-covalent heterodimeric neurotoxin crotoxin [7].

The analgesic properties displayed by crotalphine are well documented in the literature, and various receptors and ion channels have been proposed to be implicated in such properties. In particular, the antinociceptive effect of the crotalid peptide was reported to be mediated by the activation of opioid receptors in various experimental models of acute and chronic pain [7,9,10,11,12,13]. In prostaglandin E_2_ (PGE_2_)- and carrageenin-induced mechanical hyperalgesia models in rats, this antinociceptive effect was not altered by specific antagonists of mu- and delta-opioid receptors, whereas it was blocked by a specific antagonist of kappa-opioid receptors. However, crotalphine was shown to not directly activate kappa-opioid receptors, which indicates that the opioid receptor-dependent effect lies downstream of the peptide’s direct action [7]. Hence, this effect was reported to involve the activation of type-2 cannabinoid (CB_2_) receptors in the PGE_2_-induced mechanical hyperalgesia model in rats and, in turn, the local release of dynorphin A, an endogenous agonist of kappa-opioid receptors [14], and/or the Wnt (wingless-related integration site) impairment signaling pathway in the rat model of chronic constriction injury mechanical hyperalgesia [15]. In addition to kappa-opioid receptors, delta-opioid receptors were shown to be partially implicated in the antinociceptive effect of crotalphine in a rat model of cancer pain, using specific antagonists of these opioid receptors [12]. Furthermore, several lines of evidence indicate that the antinociceptive impact of crotalphine is facilitated through the activation of delta- and/or kappa-opioid receptors, which occurs as a result of the activation of the nitric oxide (NO)-dependent cyclic guanosine monophosphate (cGMP) pathway [9,11]. Additionally, this effect was suggested to also involve the ATP-sensitive K^+^ channels located in peripheral afferent neurons [11]. Moreover, type-1 transient receptor potential ankyrin (TRPA1) ion channels were also shown to be necessary for crotalid peptide-induced analgesia [16].

All these studies emphasize the fact that, despite in vivo evidence suggesting the implication of certain receptors (i.e., opioid and cannabinoid receptors) and channels (i.e., ATP-sensitive K^+^ and TRPA1 channels) in the analgesic effect of crotalphine, there is no demonstration that this crotalid peptide directly activates any of these receptors and channels. In this context, the search for additional targets of crotalphine is still relevant. Among the ten α-subunits of the mammalian Na_V_ channel (Na_V_1.1–1.10) which have been reported so far and are mainly distinguished according to their specific expression in tissues and their sensitivity (Na_V_1.1–1.4, 1.6–1.7) or insensitivity (Na_V_1.5, 1.8–1.9, 1.10) to tetrodotoxin (TTX), Na_V_1.7, 1.8, and 1.9 have been described as the major channel subtypes expressed in dorsal root ganglion (DRG) neurons, the first relay of transmission of pain signalsfrom the skin and tendons to the central nervous system [17]. In addition, during the past few years, the discovery of multiple loss- and gain-of-function mutations in the gene encoding the human (h)Na_V_1.7 channel subtype, and their association with painless and painful phenotypes, respectively, validated this subtype as a main target of analgesics [17,18,19,20]. Therefore, the aim of the present work was to test if the Na_V_1.7 channel subtype may be an additional target of crotalphine. To this purpose, the effect of this crotalid peptide was evaluated on the TTX-sensitive Na^+^ current of DRG neurons of adult mice that is largely supported by the Na_V_1.7 channel subtype [21,22].

## 2. Results

### 2.1. Effect of Crotalphine on the TTX-Sensitive Na^+^ Current

The effect of crotalphine was investigated on the TTX-sensitive Na^+^ current of DRG neurons with a cell diameter of 25.1 ± 1.4 µm (*n* = 14). The Na^+^ current of these neurons was decreased by 100 nM TTX to 8.1 ± 5.1% (*n* = 14) of the initial peak amplitude values within a few minutes. TTX was then washed out, allowing a recovery of 83.5 ± 4.5% (*n* = 14) of the current. Once the TTX sensitivity was confirmed, the effect of various concentrations of crotalphine was evaluated by measuring the peak current amplitude, recorded in the presence of the crotalid peptide, relative to its initial value.

**Figure 2:**
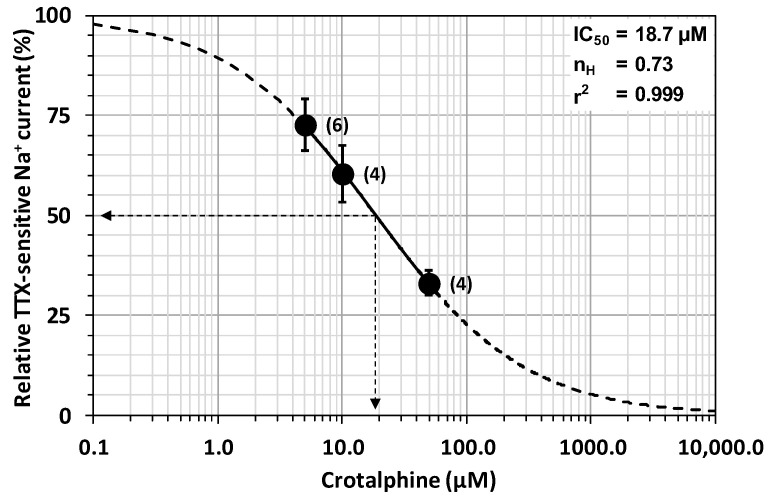
Concentration–response curve of crotalphine’s steady-state effect on the TTX-sensitive Na^+^ current. Each value of peak Na^+^ current, expressed as a percentage of that determined before peptide application, represents the mean ± S.E.M. of data obtained from four to six neurons (numbers in parentheses). The theoretical curve was calculated according to Equation (1), with IC_50_ and n_H_ values of 18.7 µM and 0.73 (r^2^ = 0.999), respectively.

Exposing neurons to standard physiological solutions containing 5 to 50 µM of crotalphine produced a decrease in Na^+^ current amplitude that was dependent on the peptide concentration and the exposure time. In particular, the concentration–response curve of crotalphine’s steady-state effect on the peak amplitude of the TTX-sensitive current revealed that the peptide concentration necessary to inhibit 50% of the current (IC_50_) and the Hill number (n_H_) were 18.7 µM and 0.73 (r^2^ = 0.999), respectively (Figure 2). The blocking effect of crotalphine on the peak current’s amplitude was stationary at 4.7 ± 1.1 min (*n* = 6), 3.7 ± 0.8 min (*n* = 4), and 3.3 ± 0.7 min (*n* = 4) after the application of 5, 10, and 50 µM of peptide, respectively. The effect of crotalphine on the TTX-sensitive current was not reversible under our experimental conditions since the peak current amplitude, which was 33.1 ± 3.0% (*n* = 4) of the initial values after the application of 50 µM of the peptide, did not markedly change by exposing the neurons to a peptide-free solution for about 10 min.

**Figure 3:**
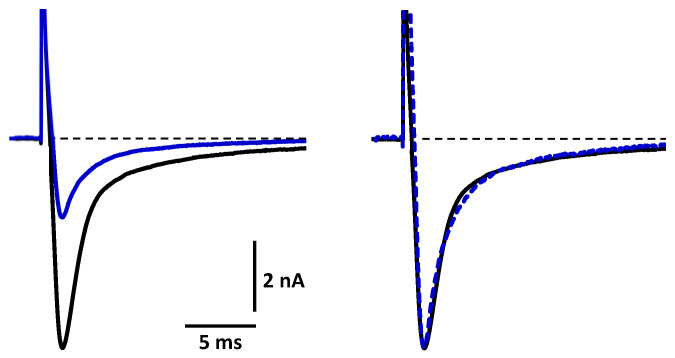
Effect of crotalphine on the activation and inactivation kinetics of the TTX-sensitive Na^+^ current. Representative traces of TTX-sensitive Na^+^ currents recorded before (in black) and after (in blue) the application of 50 µM of crotalphine. The leakage current was compensated for. On the right, the peptide peak current was normalized to that of the control to allow for a better comparison of the kinetics.

The analyses of the activation and inactivation kinetics of the TTX-sensitive current revealed that crotalphine did not affect these kinetics (Figure 3) since the time (Time_A_) and slope (Slope_A_) of current increase from 10% to 90% (activation) and the time (Time_I_) and slope (Slope_I_) of current decrease from 90% to 10% (inactivation) were not significantly different (*p* ≥ 0.622) in the absence and presence of 50 µM of the peptide (Table 1).

**Figure 4:**
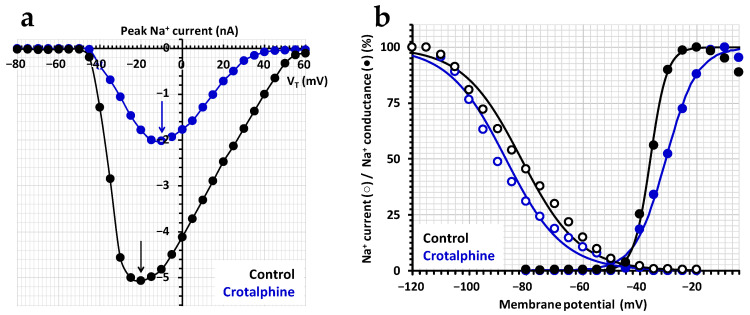
Effect of crotalphine on the activation and inactivation voltage dependencies of the TTX-sensitive Na^+^ current. (**a**) Representative current–voltage relationships before (black circles) and after (blue circles) the exposure of neurons to 50 µM of crotalphine. The black and blue arrows indicate the maximal peak Na^+^ current amplitude under the control conditions (at −20 mV) and in the presence of crotalphine (at −10 mV), respectively. (**b**) Representative steady-state inactivation–voltage (open circles) and conductance–voltage (filled circles) relationships before (black circles) and after (blue circles) the exposure of neurons to 50 µM of crotalphine. Each value is expressed as a percentage of either the maximal peak amplitude of the current at strongly negative pre-pulse voltages or the maximal conductance calculated at strongly positive test-pulse voltages. The theoretical curves correspond to data point fits according to Equations (3) and (4), with V_P50%_, k_H_, V_T50%_, and k_G_ values of −81.7 mV, 10.3 mV^−1^, −36.4 mV, and 2.9 mV^−1^ (r^2^ ≥ 0.994), respectively, for the control, and −87.2 mV, 10.2 mV^−1^, −30.9 mV, and 5.5 mV^−1^ (r^2^ ≥ 0.996), respectively, in the presence of crotalphine.

In contrast, crotalphine was shown to affect the activation and inactivation voltage dependencies for neurons exhibiting a TTX-sensitive current. Hence, the effect of 50 µM crotalphine on the current–voltage relationship of the TTX-sensitive current revealed that, in the presence of the crotalid peptide, a 10-mV less tive potential was needed to obtain the maximal peak Na^+^ current, i.e., −10 mV instead of −20 mV under the control conditions (Figure 4a). In addition, the amplitude of crotalphine’s maximal peak Na^+^ current, relative to that of the control, was decreased from 40.5 ± 1.0% between −20 and 0 mV to 8.8 ± 0.9% between 35 and 50 mV, indicating that the crotalid peptide was more efficient in blocking the current as the membrane potential became more positive. Finally, crotalphine produced only slight alterations in the steady-state inactivation– and conductance–voltage relationships, as shown in a representative experiment (Figure 4b). Hence, the test-pulse voltage corresponding to 50% of the maximal conductance (V_T50%_) of the conductance–voltage curves and the pre-pulse voltage corresponding to 50% of the maximal peak amplitude of the current (V_P50%_) of the steady-state inactivation–voltage curves were, respectively, positively and negatively shifted by about 5.5 mV along the voltage axis in the presence of 50 µM of crotalphine, with a less-than-two-fold increase in the curve slopes (k_G_ and k_H_).

**Figure 5:**
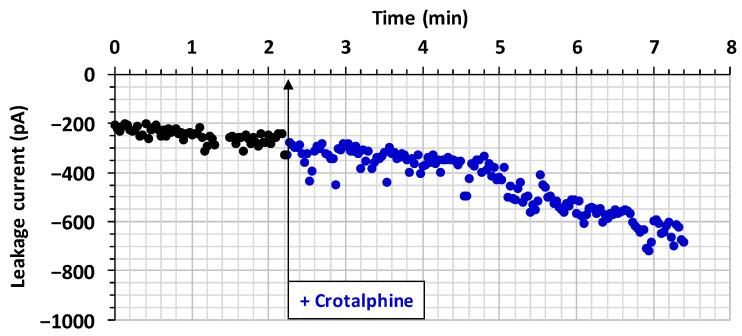
Effect of crotalphine on the leakage current. Representative experiment of the time course of crotalphine’s effect on the leakage current’s amplitude, before (black circles) and after (blue circles) the addition of 50 μM of the peptide to the standard physiological medium bathing the neurons (arrow).

While the TTX-sensitive Na^+^ current was blocked by crotalphine, an increase in the leakage current, when uncompensated, was systematically detected. Therefore, the evolution of crotalphine’s effect on the leakage current’s amplitude was studied, as a function of time, before and after the exposure of neurons to the peptide. It is worth noting that the leakage current’s amplitude was relatively stable over time, before crotalphine application. However, an increase in the leakage current’s amplitude occurred after crotalphine application, as shown in a representative experiment using 50 µM of the peptide (Figure 5). The increase in the leakage current’s amplitude was 119 ± 24% (*n* = 6), 134 ± 15% (*n* = 4), and 152 ± 23% (*n* = 4) in the presence of 5, 10, and 50 μM of crotalphine, respectively.

These results strongly suggested that the action of crotalphine also consisted in affecting cell membrane integrity by a mechanism that should alter the lipid bilayer of the cell plasma membrane. In agreement with this hypothesis, a degradation of the morphology, followed by the death, of DRG neurons was systematically observed during electrophysiological recordings, in particular in the presence of 50 μM of the peptide. In order to test this hypothesis, the effect of crotalphine (50 µM) was studied on the cell viability of cultured DRG neurons.

### 2.2. Effect of Crotalphine on Cell Viability

**Figure 6:**
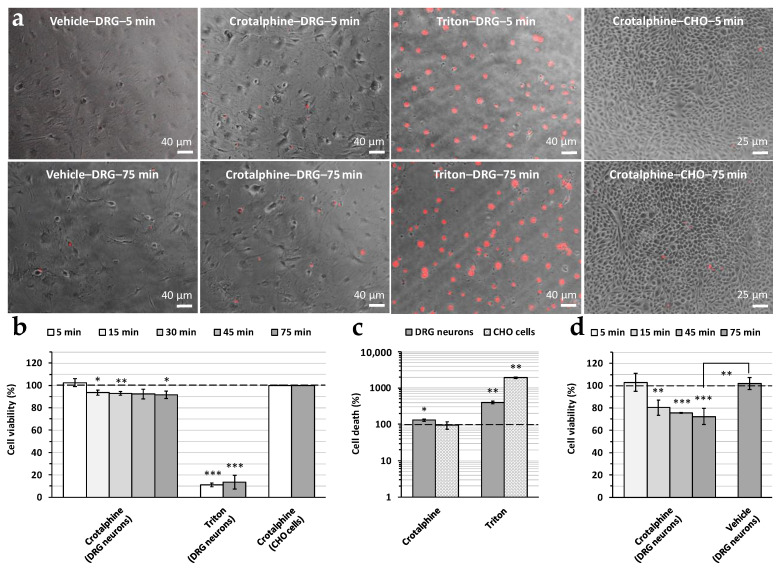
Effect of crotalphine (50 µM) on cell viability, using the propidium iodide fluorescence (**a**–**c**) or the trypan blue (**d**) assay. (**a**) Images of cultured DRG neurons and CHO epithelial cells acquired under the indicated conditions using an inverted epifluorescence microscope. The nuclei of dead cells are colored in red. (**b**) Histogram of cell viability (DRG neurons and CHO cells) in the presence of either crotalphine or triton X-100 (1%), as a function of time (from 5 to 75 min). Cell viability (number of non-fluorescent cells normalized to that of total cells) is expressed relative to that measured in the presence of the peptide vehicle, i.e., 1% of distilled water (dashed line). (**c**) Histogram of cell death (DRG neurons and CHO cells) induced by either crotalphine or triton X-100 (1%) and measured just after the end of image acquisition. The cell death (i.e., the fluorescence intensity) is expressed relatively to that measured in the presence of the peptide vehicle (dashed line). (**d**) Histogram of cell viability (DRG neurons) in the presence of either crotalphine or the peptide vehicle, as a function of time (from 5 to 75 min). Under each condition, the cell viability (number of cells having a clear cytoplasm normalized to the number of total cells) is expressed relative to that measured before peptide or vehicle addition to the cell suspension (dashed line). (**b**–**d**) Means ± S.E.M. of data obtained from three to five different experiments. *: *p* ≤ 0.0457; **: *p* ≤ 0.0069; and ***: *p* ≤ 0.0001.

Crotalphine’s effect on cell viability was studied using the propidium iodide fluorescence assay. As shown in Figure 6a,b, 50 µM of peptide induced a slight (8.5 ± 3.4%, *n* = 5) but significant decrease in the viability of the DRG neurons, compared to the vehicle (i.e., 1% of distilled water), over time (between 15 and 75 min). In contrast, a much more pronounced decrease (89.1 ± 1.7%, *n* = 4) was detected following the addition of the detergent triton X-100 (0.1%), used as a positive control, for 5–75 min to the cells. Under similar conditions, no significant peptide effect could be detected on the viability of Chinese hamster ovary (CHO) epithelial cells for 75 min. These results were confirmed by measuring, just after the end of image acquisition, the fluorescence intensity of the wells, which is a function of the number of dead cells (Figure 6c). Under these more precise conditions, a higher viability decrease in the cells in primary cultures of DRG neurons (i.e., 30.6 ± 8.7%, *n* = 3) was detected in the presence of crotalphine, compared to the peptide vehicle.

Crotalphine’s effect on cell viability was also studied using the trypan blue assay (Figure 6d). Here, again, the cell viability in primary cultures of DRG neurons (i.e., the number of cells having a clear cytoplasm normalized to that of total cells) was significantly decreased (by 27.7 ± 7.2%, *n* = 5) in the presence of 50 µM of crotalphine, over time (between 15 and 75 min), compared to the cell viability measured before the peptide’s addition to the cell suspension. Such a decrease did not occur following the addition of the peptide vehicle for 75 min.

## 3. Discussion

The present results reveal that the effects of the analgesic peptide crotalphine on the DRG neurons of adult mice consist of (1) an inhibition of the TTX-sensitive Na^+^ current with an IC_50_ of 18.7 µM, (2) a maximal increase in the leakage current’s amplitude of approximately 150%, and (3) a 30% maximal decrease in cell viability.

### 3.1. Crotalphine-Induced Blocking of Na_V_1.7 Channel Subtype

The fact that crotalphine inhibits the TTX-sensitive Na^+^ current of adult mouse DRG neurons strongly suggests that the peptide is efficient in blocking the Na_V_1.7 channel subtype, a genetically validated pain target [17,18,19,20]. Indeed, this channel subtype has been reported to be responsible for most of the TTX-sensitive current not only in mouse (83%) but also in rat (78%) and human (75.5%) DRG neurons [21,22]. Moreover, exposing DRG neurons to 10 nM of the tarantula venom peptide protoxin-II (ProTx-II), a well-known selective inhibitor of the Na_V_1.7 channel subtype IC_50_ = 0.3–1.6 nM, for hNa_V_1.7 vs. 16 nM for hNa_V_1.1 and over 25 nM for other Na_V_ channel subtypes (hNa_V_1.2–1.6 and hNa_V_1.8) [22,23,24] consistently produced a 79.2 ± 4.8% (*n* = 3) decrease in the TTX-sensitive Na^+^ current amplitude (unpublished data). Furthermore, the crotalphine-induced inhibition of the TTX-sensitive Na^+^ current occurs without any marked change in the current activation and inactivation kinetics, but the crotalid peptide is more efficient in blocking the current as the membrane potential becomes more positive. These results indicate that crotalphine may act as a Na_V_1.7 pore blocker, i.e., by obstructing the ion-conducting pore, and as a gating modifier, i.e., by altering the voltage behavior of the channel [25].

Until now, various receptors have been proposed to be implicated in the analgesic properties of the crotalid peptide. The activation of opioid receptors, notably, that of the kappa-opioid receptor, was first reported to mediate, in vivo, the crotalphine-induced antinociceptive effect in murine experimental models of acute and chronic pain when the peptide was administered to animals by oral, intravenous, or intraplantar routes [7,10]. This in vivo effect was later confirmed by in vitro experiments performed on PGE_2_-sensitized primary cultures of adult rat DRG neurons, showing not only the efficiency of crotalphine (at 1 µM) in activating kappa-opioid receptors, but also the involvement of the NO-dependent cGMP pathway in this activation [9]. It is worth noting that the in vivo demonstration of the implication of ATP-sensitive K^+^ channels in the crotalphine-induced activation of opioid receptors [11] has not been confirmed in vitro until now. More recently, the peptide was reported to produce the activation of peripheral CB_2_ receptors and, in turn, the local release of dynorphin A, an endogenous agonist of kappa-opioid receptors, both in vivo in the PGE_2_-induced hyperalgesia rat model and in vitro (at 2.6 nM and 1 µM) in paw skin tissues obtained from naïve and PGE_2_-pretreated rats [14]. In addition, crotalphine was shown to induce analgesia in a rat model of neuropathic pain (i.e., chronic constriction injury of the animal’s sciatic nerve) by down-regulating the Wnt signaling pathway (which contributes to withdrawal pain symptoms from opioid receptor activation), an effect mediated, at least in part, by CB_2_ receptor activation [15].

The effects of crotalphine on the ion channels involved in peripheral pain were poorly investigated, and, to the best of our knowledge, only one study had reported such effects until now. Hence, it was demonstrated that the transient and partial activation with the subsequent long-lasting desensitization of TRPA1 ion channels, produced in vitro by the crotalid peptide (1–10 µM), is essential for its in vivo antihyperalgesic action [16]. In this study, monitoring agonist-evoked intracellular calcium or plasma membrane potential revealed that the activity of nicotinic acetylcholine receptors (α7 and α3β2/β4 nAChRs), as well as that of voltage-sensitive calcium (Ca_V_1.3 and Ca_V_2.2) and sodium (Na_V_1.7 and Na_V_1.8) channel subtypes, was unchanged by crotalphine (10 µM). In addition, no effect of the crotalid peptide (10 µM) was detected on the compound action potential latency and amplitude of electrically stimulated C- and A-fibers of isolated mouse saphenous nerves. These results are not in disagreement with those of the present study, showing that 10 µM of crotalphine produced a 40% mean reduction in the TTX-sensitive Na^+^ current of mouse DRG neurons, if one takes into account (i) that Bressan et al. [16] used indirect approaches to evaluate the functioning of the Na_V_1.7 channel subtype and (ii) that a non-linear relationship exists between the percentages of the peak Na^+^ current and action potential inhibition. In particular, a decrease of 50% in the peak Na^+^ current will not be associated with a marked decrease in the peak action potential but, instead, with a marked decrease in the maximal rate of its rising phase [26].

In any cases, it is unlikely for the Na_V_1.7 channel subtype to be the primary target of crotalphine, considering the marked difference between the concentrations needed in vitro to block this channel subtype, i.e., micromolar range (present study), and the doses at which the crotalid peptide has been reported to exert, in vivo, its analgesic effects when administered to animals by the intraplantar route, i.e., 0.04 pmol/paw [7]. However, natural peptides are notorious for having several targets, and nature executes things sufficiently well so that these peptides act synergistically on several targets to reach an efficient physiological response. Therefore, it is likely that crotalphine has a plurality of pharmacological targets that may work together to contribute to the well-known analgesic effect of the rattlesnake venom peptide.

### 3.2. Crotalphine-Induced Decrease in Cell Viability

Another effect of crotalphine is the increase in the leakage current’s amplitude (of approximately 120 and 150% in the presence of 5 and 50 µM, respectively), systematically detected in the present study. It is likely that the increased leakage current amplitude results from interactions between the crotalid peptide and cell plasma membrane components that may affect cell membrane integrity. In agreement with this hypothesis, a decreased viability of DRG neurons (up to 30%) is observed in the presence of crotalphine (50 µM). The fact that, under similar conditions, no significant peptide effect could be detected on the viability of CHO cells is not surprising if one takes into account that the plasma membrane’s composition may vary from a cell type to another, especially between neuronal and non-neuronal cells. It is worth noting that, despite the addition of cytosine β-D-arabinofuranoside to the medium (see Section 5), astrocyte proliferation still occurred in the primary cultures of DRG neurons. Although astrocytes and DRG neurons could be easily distinguished, based on their cell morphology, for the propidium iodide assay, using the ImageJ 1.53g software, it is likely that the cell death of both astrocytes and DRG neurons was included when the fluorescence intensity of the wells was measured at the end of the propidium iodide experiments, using a microplate reader, and with the trypan blue assay, using a cell counter. This probably explains the increase in the mean percentage of cell death from 8.5% to 30.6% and 27.7%, respectively.

To the best of our knowledge, such an effect of the crotalid peptide on cell plasma membrane constituents has never been described in the literature. The simplest explanation is that crotalphine interacts also with lipid membrane constituents since interactions between gating modifier toxins and the lipid membranes surrounding the channel have been previously evidenced [25,27]. It is therefore likely that, in addition to the Na^+^ channel subtype Na_V_1.7, the crotalid peptide targets also a second cell plasma membrane component. Further experiments are still needed to identify this second membrane target of crotalphine. It is worth noting that crotamine, the most abundant protein in the *Crotalus durissus terrificus*’s venom [8], has been reported to both (i) alter the functioning of ionic channels [initially, Na_V_ channels, and, later, voltage-sensitive potassium (K_V_) channels at high and low concentrations, respectively] and (ii) decrease the viability of certain cells [28,29,30,31,32].

## 4. Conclusions

Crotalphine is shown, for the first time, to inhibit the TTX-sensitive Na^+^ channels of mouse DRG neurons, i.e., the Na^+^ channel subtype Na_V_1.7. Although this action may contribute to the well-known analgesic properties of the rattlesnake venom peptide, it is unlikely for this channel subtype to be the primary target of the crotalid peptide. In addition, although less efficiently, crotalphine decreases cell viability, likely by interacting with a second cell plasma membrane component. Therefore, the Na^+^ channel subtype Na_V_1.7 and the other cell plasma membrane component, still unidentified, should be added to the list of potential crotalphine targets. An interesting future research effort on the peptide targets and mechanisms of action would involve testing whether crotalphine activates the TWIK-related K^+^ (TREK) channels well known to interact with membrane lipids and be involved in acute and long-lasting nociception [33,34]. Finally, it is important to note that the clinical aspect of snake bites is a good indicator for the possible development of analgesics based on the venom components [2,13].

## 5. Materials and Methods

### 5.1. Chemical Synthesis and Folding of Crotalphine

Fmoc-amino acids and 2-(6-chloro-1H-benzotriazole-1-yl)-1,1,3,3-tetra-methylaminium hexafluorophosphate (HCTU) were obtained from Novabiochem (Darmstadt, Germany). The resins and all the peptide synthesis-grade reagents [N-methylpyrrolidone (NMP), N-methylmorpholine (NMM), dichloromethane, piperidine, trifluoroacetic acid (TFA), anisole, thioanisole, and triisopropylsilane] were obtained from Sigma-Aldrich (Saint-Quentin Fallavier, France).

The synthesis of crotalphine, i.e., the 14-amino acid sequence of PCA-F-S-P-E-N-C-Q-G-E-S-Q-P-C, where PCA is a pyroglutamic acid (see Figure 1) [7], was performed on a prelude synthesizer (Protein Technologies Inc., Tucson, AZ, USA) at a 25 µmoles scale using a 10-fold excess of Fmoc-amino acid relative, respectively, to the resin loading Fmoc-Cys(Trt)-wang-LLresin (0.33 mmol/g). Amino acids were coupled twice for 10 min using 1:1:2 amino acid/HCTU/NMM in NMP. After the incorporation of each residue, the resin was acetylated for 5 min using a 50-fold excess of a mixture of acetic anhydride and NMM in NMP. Fmoc deprotection was performed twice for 3 min using 20% piperidine in NMP, and 30 s NMP top washes were performed between deprotection, coupling, and acetylation. The final reduced peptide was obtained after treatment with trifluoroacetic acid and scavengers.

The disulfide bond between ^7^Cys and ^14^Cys was formed by obtaining oxidized crotalphine by air oxidation in an ammonium carbonate buffer (0.1 M, pH 8) and a peptide concentration of 0.1 mg/mL. After 48 h at room temperature, the reaction was stopped by acidification, and the peptide was purified by HPLC using an X-Bridge BHE C18-300-5 semi-preparative column (250 × 10 mm) or analytical column (250 × 4.6 mm) (Waters SAS, Saint-Quentin-en-Yvelines, France). The flow rate was 4 mL/min, and a gradient of 0–50% solvent B into A for 50 min [solvent A (H_2_O/TFA 0.1%) and solvent B (acetonitrile/TFA 0.1%)] was used. The purity rate of the purified peptide was checked by mass spectrometry using ESI-MS (Bruker France, Wissembourd, France). The calculated and found molecular weights were 1534.4 and 1533.8, respectively.

### 5.2. Toxins

Lyophilized synthetic crotalphine (1 mg, purity rate > 97%) was dissolved in distilled water to give a stock solution of 5 mM, and TTX citrate (molecular weight of 319.27, purity rate > 98%; Sigma-Aldrich) was dissolved in a phosphate-buffered saline (PBS X-1) solution with 10% phosphate buffer added to provide stock solutions of 2.85 mM. The stock solutions were stored at −20 °C. Prior to the experiments, successive dilutions were performed in the adequate media to give the final toxin concentrations indicated in the text.

### 5.3. Cultures of CHO Epithelial Cell Line and Primary Cultures of DRG Neurons

The CHO epithelial cell line was cultured as previously described [35]. Primary cultures of DRG neurons were performed using adult female Swiss mice (*Mus musculus*, 8–12 weeks of age and 25–32 g body weight), purchased from Janvier Elevage (Le Genest-Saint-Isle, France) and acclimatized at the CEA animal facility for at least 48 hr before the experiments. All experimental procedures on mice were permitted by the Animal Ethics Committee of the CEA and the French General Directorate for Research and Innovation (project APAFIS#26651-2020072011192542v1, authorized for E.B. on 20 July 2020 for 5 years). After mouse anesthesia (by 2.0–2.5% isoflurane inhalation) and euthanasia (by cervical vertebrae dislocation), the DRG neurons were dissected and placed in iced Ham’s F-12 medium (Sigma-Aldrich) and enzymatically dissociated with type-IA collagenase (2 mg/mL; Sigma-Aldrich) and dispase (5 mg/mL; Gibco, Thermo Fisher Scientific, Villebon-sur-Yvette, France). The neurons were cultured under standard conditions, as previously described [36]. One day later, cytosine β-D-arabinofuranoside (2 µM; Sigma-Aldrich) was added to the medium to inhibit astrocyte proliferation. The experiments were carried out within 4 to 8 days after neuron dissociation.

### 5.4. Electrophysiological Recordings

Prior to the electrophysiological recordings, the DRG neurons plated on coverslips were transferred to 35 mm Petri dishes filled with a standard physiological medium of the following composition for a minimum of 30 min at 37 °C: 134 mM NaCl, 3 mM KCl, 1 mM CaCl_2_, 1 mM MgCl_2_, 10 mM D-glucose, 10 mM tetraethylammonium chloride (TEA), 0.1 mM CdCl_2_, and 10 mM N-2-hydroxyethylpiperazine-N’-2-ethanesulfonic acid (HEPES) (pH 7.35, adjusted with NaOH; 302 mosm). Then, the neurons were placed in the recording bath filled with the standard physiological medium. This medium was selected as the control because it complied with the physiological external concentrations of Na^+^, K^+^, Ca^2+^, Mg^2+^, and Cl^−^ ions, as well as with the physiological external pH (maintained stable using the HEPES buffer (Sigma-Aldrich)), and contained D-glucose to maintain the cell metabolism, TEA to block the K_V_ channels, and cadmium (CdCl_2_) to inhibit the calcium channels.

Whole-cell patch-clamp experiments were performed using a MultiClamp 700B integrating patch-clamp amplifier and the pClamp10.6 software (Molecular Devices, Sunnyvale, CA, USA). The signals, acquired at a 4 kHz sample rate, were filtered at 2 kHz with a low-pass Bessel filter and digitized with the aid of a computer equipped with an analog-to-digital converter (Digidata-1440A model; Molecular Devices). The patch-clamp pipettes were filled with a medium composed of 90 mM CsCl, 40 mM CsMeSO_3_, 10 mM NaCl, 2 mM MgCl_2_, 2 mM ethylene glycol-bis(β-aminoethyl ether)-N,N,N′,N′-tetraacetic acid (EGTA), 4 mM Na_2_ATP, and 10 mM HEPES (pH 7.32, adjusted with CsOH; 292 mosm) and had a resistance of 2.8 ± 0.1 MΩ (mean ± S.E.M., *n* = 14) in the standard physiological medium. A fast solution application system allowed us to change the solution (standard physiological medium supplemented with a given toxin concentration or not) around the recorded neuron within a few seconds. The toxin’s effect was evaluated by perfusing the neurons with the standard physiological medium containing various concentrations of a given toxin or by directly adding a toxin solution of a given concentration (maximal volume of 20 μL) to the standard physiological solution bathing the cells (1 mL volume). To obtain information on the reversibility of the toxin’s effect, the neurons were perfused with a standard physiological solution devoid of toxin. The experiments were carried out at a constant room temperature (20 °C).

The neurons were maintained at a holding potential of −60 mV, and the currents were elicited at a frequency of 0.5 Hz by 50 ms test-pulses to −20 mV preceded by 1 s pulses to −100 mV. Under the control conditions, the amplitude of the leakage current (before being compensated) was −149.07 ± 33.38 pA (mean ± S.E.M., *n* = 14 neurons) and represented less than 4% of that of the peak Na^+^ current. The current–voltage relationships were obtained by varying the test-pulses from −80 to +60 mV in 5 mV increments, while the steady-state inactivation–voltage relationships were obtained by changing the pre-pulses from −120 to −20 mV in 5 mV increments.

### 5.5. Cell Viability Assays

Cell viability was quantified by propidium iodide and trypan blue dye exclusion assays. For the propidium iodide assay, DRG neurons or CHO cells, cultured in 24-well plates, were treated with 50 µM crotalphine, 1% triton-X100 (Sigma-Aldrich), or an equivalent volume of distilled water (crotalphine vehicle) for durations ranging from 5 to 75 min, in the presence of 10 µg/mL propidium iodide (BioLegend Europe BV, Amsterdam, The Netherlands). Under each condition, the cells were imaged using an inverted epifluorescence microscope (Eclipse Ti-U; Nikon, Tokyo, Japan) equipped with a Nikon Digital Sight DS-Fi2 camera and the NIS-element 3.2.6 software (Nikon). The percentage of viable cells (i.e., the number of non-fluorescent cells relative to the number of total cells) was determined using the ImageJ 1.53g software (National Institutes of Health, Bethesda, MD, USA). At the end of the experiments, the total fluorescence of each well (function of the amount of propidium iodide-positive dead cells) was measured using a CLARIOstar^®^ Plus microplate reader (BMG Labtech, Champigny-Sur-Marne, France).

For the trypan blue assay, DRG neurons were treated as for the propidium iodide assay, but in a suspension. The percentage of viable cells (i.e., the number of cells having a clear cytoplasm relative to the number of total cells) was calculated before and from 5 to 75 min after crotalphine addition or before and 75 min after vehicle addition, using the Gibco™ trypan blue stain (0.4%) and the TC10™ Automated Cell Counter (Bio-Rad, Marnes-la-Coquette, France), according to the manufacturer’s protocol.

### 5.6. Data and Statistical Analyses

The cell diameter (d) was determined from the cell membrane capacity (Cm in Farads) by assuming that the cell is a sphere whose area (Sm in m^2^) is Πd^2^ and that the cell membrane’s area is directly proportional to the cell membrane’s capacity (Sm = Cm/c, where c represents the specific membrane capacity, which does not markedly vary from one cell type to another and is, on average, 10^−2^ F/m^2^).

The concentration–response relationship was established by plotting the peak current amplitude, measured in the presence of crotalphine (It) and expressed as a percentage of the value obtained before peptide application (Ic), against the crotalphine concentration ([crotalphine]). The theoretical concentration–response curve was calculated from a typical sigmoid non-linear regression through data points (correlation coefficient = r^2^), according to Hill’s equation (GraphPad Prism 5 software):

It/Ic = 1/[1 + ([crotalphine]/IC_50_) ^nH^], (1)

where IC_50_ is the peptide concentration necessary to inhibit 50% of the current, and n_H_ is Hill’s number, i.e., the slope of the curve indicating the number of bound peptide molecules per channel.

The current kinetics were evaluated by calculating the following four activation and inactivation parameters: the slope (Slope_A_) and time (Time_A_) of current increase from 10% to 90% (activation), and the slope (Slope_I_) and time (Time_I_) of current decrease from 90% to 10% (inactivation).

The conductance (g) was calculated according to the following equation:

g = I/(V_T_ − V_Na_), (2)

where I is the peak current amplitude, V_T_ is the test-pulse voltage, and V_Na_ is the equilibrium potential of Na^+^ ions. The conductance–voltage relationships were established by plotting the conductance, expressed as a percentage of the maximal conductance (g_max_) calculated at large, positive test-pulses, as a function of the test-pulse voltage. The theoretical curves correspond to data point fits, according to Boltzmann’s equation (GraphPad Prism 5 software):

g/g_max_ = 1 − [1/(1 + exp ((V_T_ − V_T50%_)/k_G_))], (3)

where V_T50%_ is the test-pulse voltage corresponding to 50% of the maximal conductance, and k_G_ is the slope of the curve.

Steady-state inactivation–voltage relationships were established by plotting the peak current amplitude, expressed as a percentage of the maximal amplitude (I_max_) recorded in response to large, negative pre-pulses, as a function of the pre-pulse voltage (V_P_). The theoretical curves correspond to data point fits, according to Boltzmann’s equation:

I/I_max_ = 1/[1 + exp ((V_P_ − V_P50%_)/k_H_)], (4)

where V_P50%_ is the pre-pulse voltage corresponding to 50% of the maximal peak amplitude of the current and k_H_ is the slope of the curve.

The data are expressed as the means ± standard error of the mean (S.E.M.) of *n* different experiments. The statistical comparison of the values was carried out using the parametric two-tailed Student *t*-test (either paired samples for comparison within a single population or unpaired samples for comparison between two independent populations). Differences were considered statistically significant at *p* ≤ 0.05.

## Figures and Tables

**Table 1:** Kinetic parameters of activation (Time_A_ and Slope_A_) and inactivation (Time_I_ and Slope_I_) of the TTX-sensitive Na^+^ current recorded from DRG neurons under control conditions (standard physiological solution) and in the presence of 50 µM of crotalphine (means ± S.E.M. of four neurons).

	Activation		Inactivation	
	Time_A_ (ms)	Slope_A_ (nA/ms)	Time_I_ (ms)	Slope_I_ (nA/ms)
Standard physiological solution	0.66 ± 0.06	−3.33 ± 0.76	6.30 ± 0.67	0.34 ± 0.05
Crotalphine (50 µM)	0.68 ± 0.03	−3.14 ± 0.33	6.67 ± 0.62	0.31 ± 0.02

## Data Availability

Data are contained within this article.
